# Exploring the Clinical Signatures of Cervical Dysplasia Patients and Their Association With Vaginal Microbiota

**DOI:** 10.1002/cam4.70440

**Published:** 2024-12-06

**Authors:** Liqin Cheng, Chunmei Yan, Yongxia Yang, Fanzhen Hong, Juan Du

**Affiliations:** ^1^ Department of Microbiology, Tumor and Cell Biology, Centre for Translational Microbiome Research (CTMR) Karolinska Institutet Stockholm Sweden; ^2^ The Department of Pathophysiology, School of Basic Medicine Science Central South University Changsha China; ^3^ Department of Obstetrics The Second Hospital of Shandong University Jinan China; ^4^ Department of Gynecology The Second Hospital of Shandong University Jinan China

**Keywords:** cervical dysplasia, human papillomavirus, vaginal microbiota

## Abstract

**Aims:**

The vaginal microbiota plays a crucial role in women's health, and an imbalanced vaginal microbiota is linked to various diseases, including human papillomavirus (HPV) infection. However, most available data comes from Western countries and primarily focuses on HPV infection, with only a few studies considering detailed clinical factors to explore the relationship between vaginal microbiota and the development of cervical cancer, especially in China.

**Materials and Methods:**

Our study involved 266 women, including individuals at all stages of cervical dysplasia, and healthy controls with and without HPV infection. We assessed several aspects of the vaginal environment, including vaginal microbiota composition using 16S rRNA gene sequencing, HPV infection status using the standard Roche Cobas method, pH value, age, and H_2_O_2_ levels from clinical records, and partner numbers and contraceptive methods obtained through questionnaires. The association of these clinical signatures with cervical dysplasia stages and vaginal microbiota was analyzed.

**Key Findings:**

Our findings demonstrate a significant association between vaginal microbiota and cervical dysplasia stages. Patients with cervical dysplasia and cancer showed a substantial increase in HPV 16 infection, a higher prevalence of pH > 5, a lower H_2_O_2_ level, and older ages compared to healthy individuals. Additionally, these factors influence the beta diversity of the vaginal microbiota.

**Significance:**

These results underscore the importance of considering the vaginal microbiota within the cancer microenvironment and highlight the need to integrate all available data to aid in the current diagnosis and understanding of cervical dysplasia and the cervical cancer microenvironment.

## 1 Introduction

Cervical cancer is among the most commonly diagnosed female cancers, causing over 300,000 deaths annually [1]. Numerous studies have recently investigated the association between vaginal microbiota and sexually transmitted infections, such as human papillomavirus (HPV), and related conditions like cervical dysplasia [2, 3]. In women of reproductive age, the vaginal microbiota is typically dominated by a single species of *Lactobacillus*, including *L. crispatus* , *L. iners* , *L. gasseri* , and *L. jensenii* . *Lactobacillus* species protect against pathogens through various mechanisms, such as lactic acid secretion, which lowers the environmental pH, and the production of H_2_O_2_ and bacteriocins that inhibit pathogen growth. Additionally, *Lactobacillus* species modulate the immune response [4]. Concentration of H_2_O_2_, produced by *Lactobacillus* and host cells in the cervix, has been associated with bacterial vaginosis [5, 6]. Apart from *Lactobacillus*‐dominated vaginal microbiota, some women exhibit higher diversity in their vaginal microbiota, with bacterial species dominated by *Gardnerella*, *Prevotella*, and *Sneathia*, among others. Non‐*Lactobacillus*‐dominated microbiota is commonly observed in women with bacterial vaginosis and sexually transmitted infections, but it can also occur in asymptomatic women [7, 8, 9].

Persistent infection of high‐risk oncogenic HPV subtypes, particularly HPV16 and 18, is widely recognized as a major risk factor for dysplasia and cervical lesion development [10, 11]. Several other factors, such as age, number of sexual partners, and family history, have also been reported to be associated with the development of cervical cancer [12, 13, 14]. Recent studies have demonstrated a correlation between vaginal microbiota and HPV infection [8, 15]. Moreover, a decreased abundance of *Lactobacillus* species has been observed in cases of prevalent high‐risk HPV infection [16]. Our previous studies have highlighted a strong association between HPV infection, cervical dysplasia, and a non‐*Lactobacillus*‐dominant vaginal microbiota [17]. Furthermore, risky sexual behavior has been shown to contribute to increased vaginal microbial diversity and a higher incidence of cervical cancer [18].

The composition of the vaginal microbiota is known to be influenced by various factors, including pathogen colonization, ethnicity, hormonal changes, hygiene habits, and sexual activity [19, 20, 21]. However, most available data on this topic comes from Western countries and primarily focuses on HPV infection, with only a limited number of studies exploring the interaction between vaginal microbiota and the development of cervical cancer, particularly in China [22, 23, 24]. Therefore, to provide more comprehensive information on vaginal microbiota in cervical dysplasia, considering detailed clinical factors, and comparing the data from China with other countries, we conducted a thorough investigation of the vaginal microenvironment. This investigation took vaginal microbiota composition, HPV infection status, pH value, age, H_2_O_2_ level, partner numbers, and contraceptive methods into account, and analyzed their association with cervical dysplasia stages.

## 2 Materials and Methods

### 2.1 Sample Collection

A total of 266 women aged 18–75 were recruited at the Second Hospital of Shandong University Hospital in China from 2018 to 2019. The exclusion criteria were as follows: (1) participants who had taken any antibiotics, probiotics, or immune inhibitors during the past 3 months; (2) participants with other internal or surgical diseases; (3) participants who had engaged in sexual behavior during the past 3 days; and (4) pregnant participants.

Detailed medical and gynecological histories were obtained following international guidelines, including age, HPV types, dysplasia stage based on histology and cytology, vaginal pH value, HPV vaccination status, number of sexual partners, and contraceptive method (Table S1). All samples were collected during the clinical visit by gynecologists using vaginal swabs. The swabs were immediately placed inside tubes containing 1 mL of DNA/RNA Shield (Zymo Research, USA). The samples were stored at −20°C until further DNA extraction.

All patients underwent colposcopy examinations, which were performed by one of the two gynecologists, Dr. Yongxia Yang and Dr. Chunmei Yan. Liquid‐based cytopathology tests (LCT) were conducted using the SurePath LCT kit (BD SurePath, USA), followed by the AutoCyte PREP system (Tripath Imaging, USA) for automated slide preparation and staining. Biopsies were obtained from visible lesions and histologically graded using standard procedures at the Second Hospital of Shandong University Hospital. Healthy participants were recruited from individuals visiting the hospital for regular vaginal examinations. Information on HPV types, HPV vaccination status, number of sexual partners, and contraceptive methods was collected from all healthy participants.

### 2.2 Identification of pH Level, H_2_O_2_ Level, and Fungi/Fungal Spores

The pH level, H_2_O_2_, and fungi/fungal spores were measured using the vaginitis combined detection kit, following the standard protocol (Jiangsu Yi Ruoweisheng Bioisystech Co Ltd. China). The detection was conducted on the Automated Workstation for Vaginosis Detection (bPR‐2014A, Jiangsu Yi Ruoweisheng Bioisystech Co Ltd. China). The pH measurement was classified as below 4.5, between 4.5 and 5, and above 5. For the H_2_O_2_ measurement, it was categorized as low (below 2 umol/L), intermediate (between 2 and 4 umol/L), and high (above 4 umol/L).

### 2.3 HPV Genotyping

HPV genotypes, including HPV 16, 18, and pooled oncogenic HPV 31, 33, 35, 39, 45, 51, 52, 56, 58, 59, 66, and 68, were tested using the Roche Cobas 4800 system (Roche, Switzerland) following the standard protocol.

### 2.4 DNA Extraction

DNA extraction, sequencing, and subsequent analysis were conducted using the platform provided by Shanghai Personal Biotechnology Co. Ltd. China. Briefly, DNA was extracted from the collected vaginal swab samples using the Mag‐Bind Soil DNA kit (Omega Bio‐tek, USA). The final DNA concentration and purification were determined using the Qubit 2.0 fluorometer (Life Technologies, USA). The extracted DNA was eluted in 50 μL of DNase‐free water and stored at −20°C until further library preparation and 16S rRNA gene sequencing. Nuclease‐free water served as the negative control.

### 2.5 Microbiota Sequencing

The V3‐V4 regions of the 16S rRNA gene were amplified using Illumina sequencing primer pairs 338F (5′‐ACTCCTACGGGAGGCAGCA‐3′) and 806R (5′‐GGACTACHVGGGTWTCTAAT‐3′). PCR reactions were performed in a 25 μL system, consisting of 5 μL reaction buffer (5×), 5 μL GC buffer (5×), 2 μL deoxynucleoside triphosphates (dNTPs, 2.5 mM), 1 μL of each primer (10 μM), 0.25 μL Q5 DNA polymerase, and 40 ng of DNA template. After library preparation, the finished libraries were quantified using the Quant‐iT PicoGreen dsDNA Assay Kit (Thermo Fischer Scientific, USA) and the Agilent Bioanalyzer (Agilent, USA) before being submitted for sequencing on the Illumina MiSeq platform (Illumina, USA) with the MiSeq Reagent Kit v3 (Illumina, USA).

### 2.6 Taxonomic Assignment

Microbiome bioinformatics analyses were conducted using QIIME 22019.4, following the official tutorials available at https://docs.qiime2.org/2019.4/tutorials/. In summary, the raw sequence data were demultiplexed using the demux plugin, and the primers were removed using the cutadapt plugin. The sequences were then quality trimmed, denoised, merged, and chimera removed using the DADA2 pipeline. Non‐singleton amplicon sequence variants (ASVs) were aligned using MAFFT, and phylogenetic trees were constructed using FastTree2. Taxonomy was assigned to the ASVs at the species level using the classify‐sklearn naïve Bayes algorithm with the Greengenes reference sequences. Dominant *Lactobacillus* spp., which are commonly found in vaginal microbiota, as well as bacteria associated with bacterial vaginosis (BVAB), were further annotated and validated by performing manual BLAST searches against the National Center for Biotechnology Information (NCBI) database, as previously described [7].

### 2.7 Microbiota Diversity Analysis and Statistics

The sequence table was rarefied to 17,691 reads per sample before calculating diversity metrics. Samples that exhibited a relative abundance of more than 50.00% for a particular species were defined as species‐specific dominated. Alpha diversity metrics, including Simpson, Chao 1, Shannon, Simpson, and Faith's PD, were calculated using the “qiime diversity alpha‐rarefaction” plugin in QIIME2 (2019.4). Differences in microbiota alpha diversity among different groups were assessed using the Kruskal–Wallis test, followed by Dunn's test as a post hoc test. Beta diversity metrics, specifically Jaccard and Bray–Curtis dissimilarity, were computed using the “qiime diversity core‐metrics‐phylogenetic” plugin in QIIME2 (2019.4). The dissimilarity in microbiota beta diversity was analyzed using the Permutation Multivariate Analysis of Variance (PERMANOVA) test, implemented through the “qiime diversity beta‐group‐significance” plugin in QIIME2 (2019.4). To identify differentially abundant genera as biomarker candidates among groups with different clinical statuses, LEfSe was employed with the default parameters. The results were visualized using the “ggtree” package in R, represented as a cladogram.

### 2.8 Receiver Operating Characteristic Curve (ROC Curve)

The sensitivity and specificity of the potential factors in predicting the development of dysplasia and cancer were analyzed using the area under the ROC Curve (AUC) on SPSS. Furthermore, Fisher's exact test was used to compare the percentage of pH, H_2_O_2_, age, and vaginal microbiota with HPV infection or dysplasia status. The correlation between HPV infection, pH, H_2_O_2_, age, CIN status, and different vaginal microbiota was also analyzed and compared using Fisher's exact test.

## 3 Results

### 3.1 Study Population

A total of 266 women were enrolled in this study. Vaginal samples with low DNA concentration and low reads in sequencing (*n* = 12) were excluded from all subsequent analyses. The remaining 254 samples were divided into six main groups based on their HPV status and histology grades along the cervical cancer development: healthy and HPV‐negative (*n* = 50); healthy and HPV‐positive (*n* = 64); cervical intraepithelial neoplasia grade 1 (CIN1) (*n* = 45), CIN2 (*n* = 47), CIN3 (*n* = 34); and cervical cancer (*n* = 14). Women with incomplete clinical information were excluded from the specific analyses as follows: pH value (*n* = 9), H_2_O_2_ level (*n* = 60), sexual partner numbers (*n* = 110), contraceptive methods (*n* = 13), and fungal hyphae (*n* = 2).

Out of the 254 samples with HPV genotyping information, 58 (22.83%) were HPV‐negative, 82 (32.28%) were HPV 16 positive (with six samples also positive for HPV 18), 20 (7.87%) were HPV 18 positive, and 94 were positive for other oncogenic HPV types than HPV 16 and HPV 18. Among the 245 samples that had information on the pH level in the vaginal tract, 65 (26.53%) samples had a pH below 4.5, 80 (32.65%) samples had a pH between 4.5 and 5, and 100 (40.82%) samples tested above pH 5. Out of the 254 samples with age information, 70 (27.56%) were collected from the 18–30 age group, 110 (43.31%) were from the 31–45 age group, and 74 (29.13%) were from the 45–75 age group. The H_2_O_2_ levels were classified as low in 96 (49.48%) individuals, intermediate in 73 (37.63%) individuals, and high in 25 (12.89%) individuals among the 194 participants with H_2_O_2_ level data. Only 144 out of 254 participants reported sexual partner numbers, with the majority having one partner (*n* = 126, 87.50%), and 18 participants had more than one partner. Out of the 241 participants with contraceptive information, women with menopause (*n* = 4), contraceptive pill (*n* = 2), sterilization (*n* = 13), and hysterectomy (*n* = 1) were excluded for later comparison due to the small sample size of these conditions. A total of 221 participants were analyzed, including groups without a contraceptive method (*n* = 109, 49.32%), with an intrauterine device (*n* = 36, both past and current users, 16.29%), and with a condom (*n* = 76, 34.39%). No fungal hyphae were observed in any of the tested women (*n* = 252). Only two women received the HPV vaccine, and one of them had low DNA concentrations for sequencing. All participants tested negative for fungi, considering both hyphae and spores (Table 1).

**TABLE 1:** The characteristics of all participants organized by cervical cancer progression.

	Healthy	HPV	CIN1	CIN2	CIN3	Cervical cancer	Total	*p* (Fisher's exact test)
	*N* (%)	*N* (%)	*N* (%)	*N* (%)	*N* (%)	*N* (%)	*N* (%)	
HPV								
HPV16	0 (0%)	15 (23.44%)	11 (24.44%)	24 (51.06%)	20 (58.82%)	12 (85.71%)	82 (32.28%)	< 0.001
HPV18	0 (0%)	8 (12.50%)	4 (8.89%)	6 (12.77%)	2 (5.88%)	0 (0%)	20 (7.87%)	
Other HPV	0 (0%)	41 (64.06%)	27 (60%)	15 (31.91%)	10 (29.41%)	1 (7.14%)	94 (37.01%)	
HPV negative	50 (100%)	0 (0%)	3 (6.67%)	2 (4.26%)	2 (5.88%)	1 (7.14%)	58 (22.83%)	
pH								
pH < 4.5	24 (48.98%)	17 (29.31%)	12 (26.67%)	5 (10.64%)	6 (18.18%)	1 (7.69%)	65 (26.53%)	< 0.001
pH 4.5–5	12 (24.49%)	20 (34.48%)	18 (40%)	22 (46.81%)	8 (24.24%)	0 (0%)	80 (32.65%)	
pH > 5	13 (26.53%)	21 (36.21%)	15 (33.33%)	20 (42.55%)	19 (57.58%)	12 (92.31%)	100 (40.82%)	
H_2_O_2_								
H_2_O_2_ < 2 μmol/L	3 (33.33%)	25 (49.02%)	24 (54.55%)	23 (52.27%)	12 (36.36%)	9 (69.23%)	96 (49.48%)	0.4228
H_2_O_2_ 2–4 μmol/L	4 (44.44%)	20 (39.22%)	13 (29.55%)	18 (40.91%)	14 (42.42%)	4 (30.77%)	73 (37.63%)	
H_2_O_2_ > 4 μmol/L	2 (22.22%)	6 (11.78%)	7 (15.91%)	3 (6.82%)	7 (21.21%)	0 (0%)	25 (12.89%)	
Age								
18–30	23 (46%)	17 (26.56%)	10 (22.22%)	10 (21.28%)	10 (29.41%)	0 (0%)	70 (27.56%)	0.02399
31–44	19 (38%)	27 (42.19%)	20 (44.44%)	25 (53.19%)	13 (38.24%)	6 (42.86%)	110 (43.31%)	
45–75	8 (16%)	20 (31.25%)	15 (33.33%)	12 (25.53%)	11 (32.35%)	8 (57.14%)	74 (29.13%)	
Microbiota								
*L. crispatus*	14 (28%)	13 (20.31%)	12 (26.67%)	6 (12.77%)	6 (17.65%)	1 (7.14%)	52 (20.47%)	0.6687
*L. iners*	21 (42%)	26 (40.63%)	20 (44.44%)	24 (51.06%)	15 (44.12%)	4 (28.57%)	110 (43.31%)	
*Lactobacillus* other	4 (8%)	4 (6.25%)	3 (6.67%)	4 (8.51%)	3 (8.82%)	1 (7.14%)	19 (7.48%)	
Non‐*Lactobacillus*	11 (22%)	21 (32.81%)	10 (22.22%)	13 (27.66%)	10 (29.41%)	8 (57.14%)	73 (28.74%)	

### 3.2 Higher Microbiota Diversity Was Observed Among Cervical Cancer Patients

**FIGURE 1:**
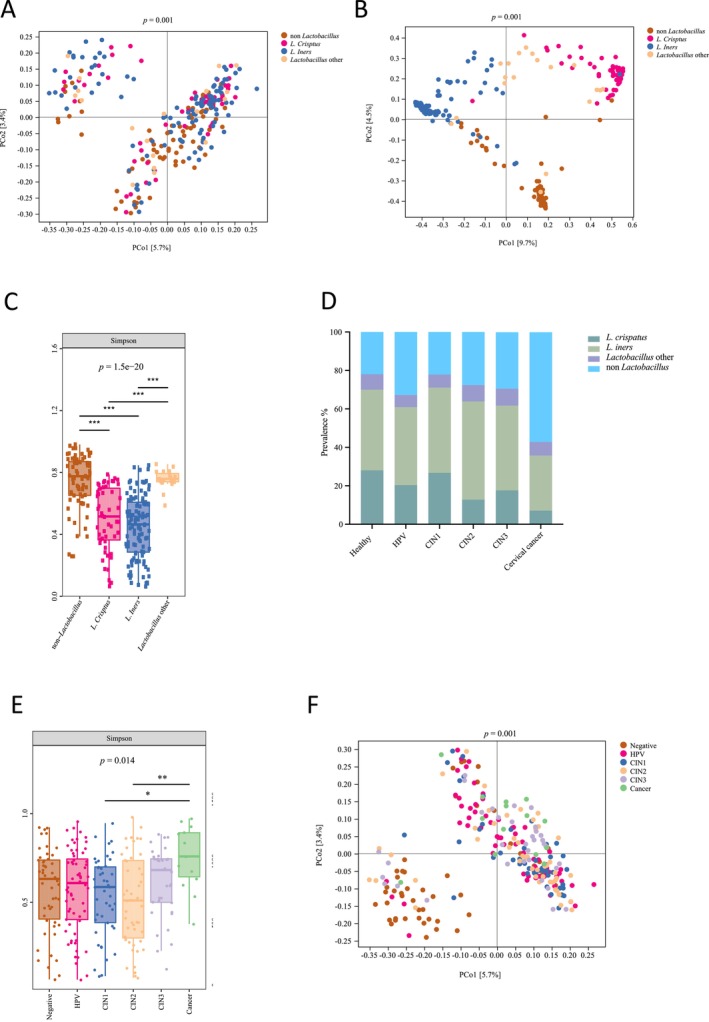
Different vaginal microbiota diversity was observed among healthy participants with and without HPV infection and cervical dysplasia and cancer patients. (A) Principal coordinates analysis (PCoA) of vaginal microbiota among women with different microbiota compositions based on Jaccard distance. (B) PCoA of vaginal microbiota among women with different microbiota composition based on Bray–Curtis distance. (C) Microbial alpha diversity analysis based on the Simpson index of women with different microbiota composition. Data were presented with median values and 1.5 times the interquartile range (IQR). Statistical significance between the groups was tested by the Kruskal–Wallis test with Dunn's test as a post hoc test.***p < 0.001. (D) The prevalence of different microbiota compositions among different groups. (E) Microbial alpha diversity analysis based on the Simpson index among different groups. Data were presented with median values and 1.5 IQR. Statistical significance between the groups was tested by the Kruskal–Wallis test with Dunn's test as a post hoc test. *p < 0.1, **p < 0.01. (F) PCoA of vaginal microbiota among different groups based on Jaccard distance.

We first evaluated the beta diversity of the vaginal microbiota samples using the Jaccard distance. Two main clusters were observed: one cluster predominantly contained samples dominated by *Lactobacillus*, while the other cluster included non‐*Lactobacillus*‐dominated samples and samples dominated by *L. crispatus* and *L. iners* (Figure 1A). To further differentiate between *Lactobacillus* species, the Bray–Curtis distance was employed to analyze the beta diversity of the vaginal microbiota. The samples dominated by *L. crispatus* and *L. iners* were clearly separated, with other samples dominated by various *Lactobacillus* species distributed in between. The non‐*Lactobacillus*‐dominated samples exhibited significant differences and formed a distinct cluster (*p* = 0.001) (Figure 1B).

Alpha diversity analysis, based on the Simpson and Shannon indices, revealed a significantly lower diversity in women belonging to the *L. crispatus* and *L. iners* ‐dominated group compared to women in the non‐*Lactobacillus*‐dominated group or other *Lactobacillus*‐dominated group (*p* = 1.5e‐20 for Simpson index, *p* = 3.3e‐18 for Shannon index) (Figure 1C and Figure S1A). This indicates that *L. crispatus* and *L. iners* have the potential to be the dominant species in the vaginal microbiota, co‐existing with fewer other species.

We then examined the association between microbiota composition and cervical cancer development. Although we observed a trend towards an increased percentage of non‐*Lactobacillus*‐dominated samples, comparing the proportions of women with different microbiota compositions in the various dysplasia and cancer groups did not reach statistical significance (*p* = 0.67) (Figure 1D). Furthermore, when analyzing the alpha diversity of the vaginal microbiota, only the cervical cancer group exhibited higher diversity compared to the CIN1 and CIN2 groups (*p* = 0.014 for Simpson index, *p* = 0.0094 for Shannon index, and *p* = 0.011 for Faith's PD) (Figure 1E and Figure S1B).

We further compared the beta diversity of the vaginal microbiota throughout the progression of cervical cancer. Figure 1F illustrates that most samples from healthy individuals with HPV negativity clustered together in the *Lactobacillus*‐dominated region, while the other cluster encompassed samples from HPV‐infected individuals, dysplasia cases, and cervical cancer patients (*p* = 0.001). This cluster also included a substantial number of non‐*Lactobacillus*‐dominated samples (Figure 1A and Figure 1F). Notably, the samples from cervical cancer patients predominantly clustered in the non‐*Lactobacillus*‐dominated and *L. iners* ‐dominated regions, whereas the majority of healthy HPV‐negative samples clustered in the area where *L. crispatus* ‐dominated and *L. iners* ‐dominated vaginal microbiota were prevalent (Figure 1B and Figure S1C).

**FIGURE 2:**
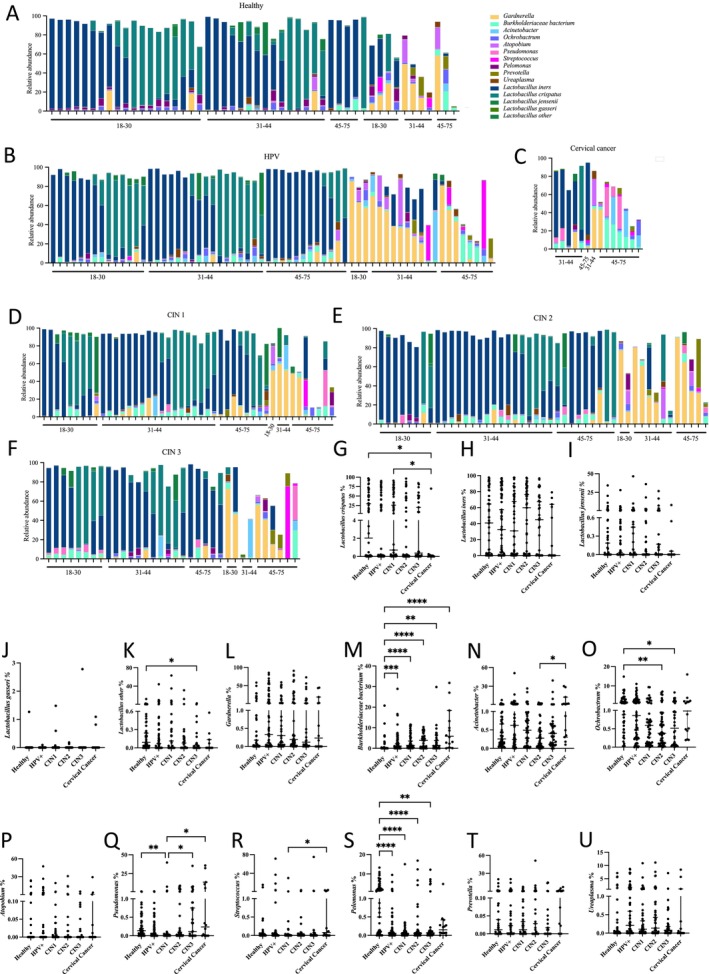
Vaginal microbiota composition at the genus/species level was grouped by dysplasia stage and age. (A–F) *Lactobacillus* species and the top 10 bacteria genus were presented based on their relative abundance. Only the bacteria with over 1.00% mean relative abundance in all the samples, *Lactobacillus* species with more than 10.00% of reads in any sample, and non‐*Lactobacillus* genera with over 30.00% of reads in any sample were included. The age information of each sample was labeled. (G–U) The relative abundance of the listed bacteria among healthy individuals and dysplasia and cancer patients was presented. Each dot on the graph represents the proportion of one individual. The data were presented with median values with a 95.00% confidence interval. Statistical significance between the groups was tested by the Kruskal–Wallis test with Dunn's test as a post hoc test. *p < 0.05, **p < 0.01, ***p < 0.001, ****p < 0.0001.

Figure 2 depicts the detailed vaginal microbiota composition of each participant categorized by dysplasia grades and age groups (Figure 2A–F). Overall, most women exhibited dominance of *Lactobacillus iners* and/or *Lactobacillus crispatus* , which typically accounted for over 90.00% of the total reads in each sample, irrespective of the dysplasia grades. Non‐*Lactobacillus*‐dominant vaginal microbiota displayed a variety of anaerobic and aerobic bacteria, including *Gardnerella*, *Burkholderiaceae bacterium*, and *Acinetobacter* (Figure 2A–F).

Furthermore, although not statistically significant, we observed a trend of progressively increasing prevalence of non‐*Lactobacillus*‐dominated vaginal microbiota (22.00% to 57.10%) and decreasing prevalence of *L. crispatus* ‐dominated vaginal microbiota (28.00% to 7.10%) from CIN1 to cervical cancer (Table 1). We also examined the proportions of the top 15 abundant bacteria in different groups. Our findings revealed an incremental level of *Burkholderiaceae bacterium* from the healthy group to the cervical cancer group, along with a reduction in levels of *L. crispatus* and *Pelomonas* from the healthy group to the cervical cancer group (Figure 2G–U). Other trends were less pronounced, with slightly lower levels of *Ochrobactrum* in the CIN2 and CIN3 groups compared to the healthy group (Figure 2O) and decreased levels of *Acinetobacter* in CIN2 and *Streptococcus* in CIN1 compared to the cancer samples (Figure 2N,R).

### 3.3 The Prevalence of HPV 16 Increased Along the Cervical Dysplasia Stages

**FIGURE 3:**
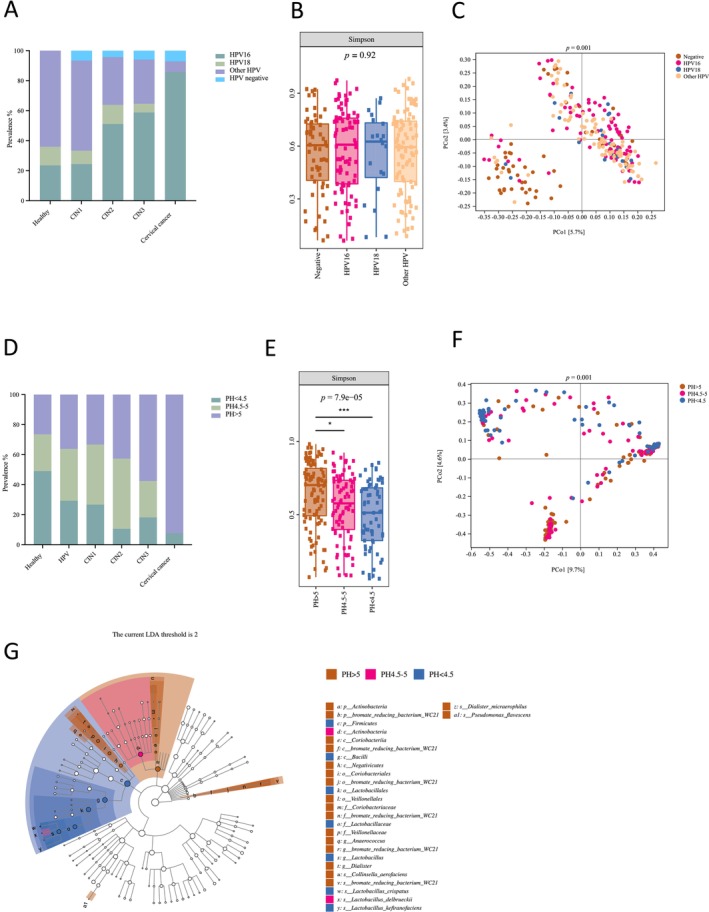
HPV infection and pH value are associated with cervical cancer progression and vaginal microbiota diversity. (A) The HPV prevalence in healthy individuals with and without HPV infection and dysplasia and cancer patients. (B) Microbial alpha diversity based on the Simpson index of women with different HPV infection statuses. Data were presented with median values and 1.5 times the IQR. Statistical significance between the groups was tested by the Kruskal–Wallis test with Dunn's test as a post hoc test. (C) PCoA of vaginal microbiota among women with different HPV infection status based on Jaccard distance. (D) The percentage of women with different pH values across all the different groups. (E) Microbial alpha diversity based on the Simpson index among women with different pH values. Data were presented with median values and 1.5 times IQR. Statistical significance between the groups was tested by the Kruskal–Wallis test with Dunn's test as a post hoc test. *p < 0.05, ***p < 0.001. (F) PCoA of vaginal microbiota among women with different pH values based on Bray‐Curtis distance. (G) LEfSe analysis compared the significantly different expressed microbes among the groups with different pH values. Only taxa with linear discriminant analysis (LDA) scores over 2 were presented.

Overall, HPV was detected in over 90.00% of women with dysplasia and cervical cancer samples. Out of HPV‐positive cases, the prevalence of HPV 16 increased from 23.43% (15/64) in healthy individuals to 24.44% (11/45) in the CIN1 group, 51.06% (24/47) in the CIN2 group, and continued to accelerate to 58.82% (20/34) in CIN3 group and can be detected in 85.71% (12/14) cases among cervical cancer patients. As the number of HPV 16 cases increased, there was a significant decrease in the prevalence of other oncogenic HPV types, excluding HPV 16 and HPV 18, among dysplasia and cancer cases, with 64.06% (41/64) in healthy HPV‐positive individuals, 60.00% (27/45) in CIN1, 31.91% (15/47) in CIN2, 29.41% (10/34) in CIN3, and 7.14% (1/14) in cervical cancer (Figure 3A). A significant association between HPV infection and cervical cancer development was demonstrated (*p* < 0.001). When comparing the microbiota diversity among women with different HPV infection statuses, no significant differences were found (Figure 3B, Figure S2A, and Table 2). However, the beta diversity of the vaginal microbiota indicated that most HPV‐uninfected samples clustered together in the *Lactobacillus*‐dominated region, while samples from women infected with HPV 16 and other HPV types clustered in the other corner where *L. iners* and non‐*Lactobacillus* vaginal microbiota samples were prevalent (*p* = 0.001) (Figures 1 and 3C, Figure S2B).

**TABLE 2:** The characteristics of all participants organized by vaginal microbiota composition.

	*L. crispatus*	*L. iners*	*Lactobacillus* other	Non*‐Lactobacillus*	Total	*p* (Fisher's exact test)
	*N* (%)	*N* (%)	*N* (%)	*N* (%)	*N* (%)	
HPV						
HPV16	14 (26.92%)	33 (30%)	7 (36.84%)	28 (38.36%)	82 (32.28%)	0.6562
HPV18	3 (5.77%)	9 (8.18%)	3 (15.79%)	5 (6.85%)	20 (7.87%)	
Other HPV	20 (38.46%)	43 (39.09%)	5 (26.32%)	26 (35.62%)	94 (37.01%)	
HPV negative	15 (28.85%)	25 (22.7%)	4 (21.1%)	14 (19.2%)	58 (22.8%)	
pH						
pH < 4.5	29 (59.18%)	29 (27.36%)	6 (33.33%)	1 (1.39%)	65 (26.53%)	< 0.001
pH 4.5–5	12 (24.49%)	42 (39.62%)	5 (27.78%)	21 (29.17%)	80 (32.65%)	
pH > 5	8 (16.33%)	35 (33.02%)	7 (38.89%)	50 (69.44%)	100 (40.82%)	
H_2_O_2_						
H_2_O_2_ < 2 μmol/L	17 (47.22%)	37 (44.05%)	5 (35.71%)	37 (61.67%)	96 (49.48%)	< 0.001
H_2_O_2_ 2–4 μmol/L	6 (16.67%)	43 (51.19%)	3 (21.43%)	21 (35%)	73 (37.63%)	
H_2_O_2_ > 4 μmol/L	13 (36.11%)	4 (4.76%)	6 (42.86%)	2 (3.33%)	25 (12.89%)	
Age						
18–30	23 (44.23%)	30 (27.27%)	5 (26.32%)	12 (16.44%)	70 (27.56%)	< 0.001
31–44	22 (42.31%)	50 (45.45%)	13 (68.42%)	25 (34.25%)	110 (43.31%)	
45–75	7 (13.46%)	30 (27.27%)	1 (5.26%)	36 (49.32%)	74 (29.13%)	
CIN						
Healthy	14 (26.92%)	21 (19.09%)	4 (21.05%)	11 (15.07%)	50 (19.69%)	0.6602
HPV	13 (25%)	26 (23.64%)	4 (21.05%)	21 (28.77%)	64 (25.20%)	
CIN1	12 (23.08%)	20 (18.18%)	3 (15.79%)	10 (13.70%)	45 (17.72%)	
CIN2	6 (11.54%)	24 (21.82%)	4 (21.05%)	13 (17.81%)	47 (18.50%)	
CIN3	6 (11.54%)	15 (13.64%)	3 (15.79%)	10 (13.70%)	34 (13.39%)	
Cervical cancer	1 (1.92%)	4 (3.64%)	1 (5.26%)	8 (10.96%)	14 (5.51%)	

### 3.4 An Elevated Vaginal pH Was Correlated With Advanced Dysplasia Stages and Increased Microbiota Diversity

**FIGURE 4:**
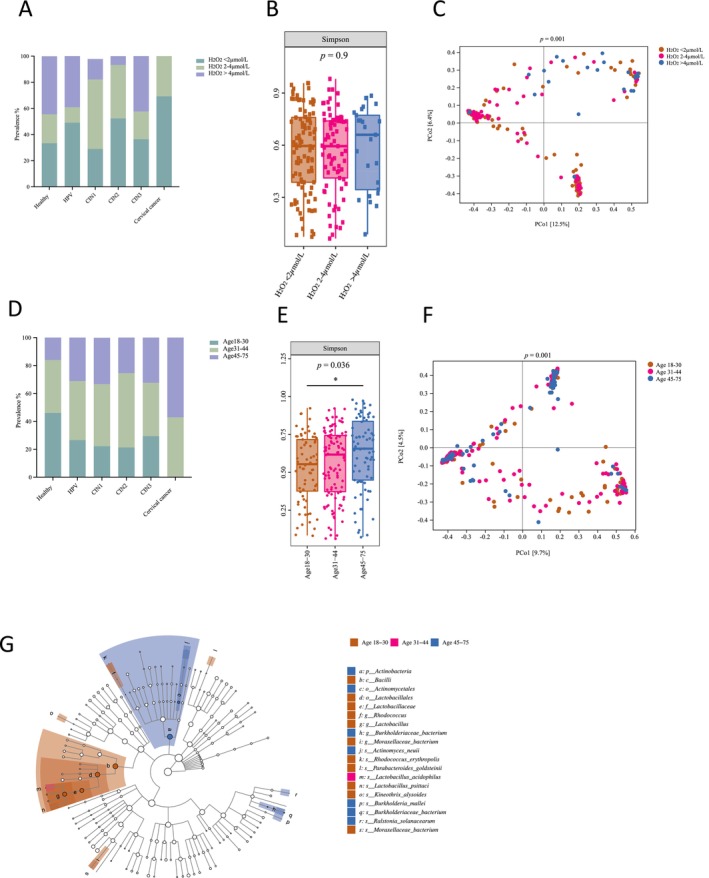
H_2_O_2_ levels and age are associated with vaginal microbiota. (A) The percentage of different H_2_O_2_ levels among healthy individuals with and without HPV infection and dysplasia and cancer patients. (B) Microbial alpha diversity among women with different vaginal H_2_O_2_ levels based on the Simpson index. The data were presented with median values and 1.5 times the IQR. Statistical significance between the groups was tested by the Kruskal–Wallis test with Dunn's test as a post hoc test. (C) PCoA of vaginal microbiota presented by different H_2_O_2_ levels based on Bray–Curtis distance. (D) The proportion of women in different age groups among different groups. (E) Microbial alpha diversity of different age groups based on the Simpson index. The data were presented with median values and 1.5 times the IQR. Statistical significance between the groups was tested by the Kruskal–Wallis test with Dunn's test as a post hoc test.*p < 0.05. (F) PCoA of vaginal microbiota among different age groups based on Bray–Curtis distance. (G) LEfSe analysis showed significantly different expressed microbes among different age groups. Only taxa with linear discriminant analysis (LDA) scores over 2 were presented.

As the cancer stages advanced, we observed a significant association between elevated vaginal pH and lesion grade (*p* < 0.001) (Table 1). The proportion of women with a pH greater than 5 gradually increased from 26.53% in the healthy group to 36.21% in the healthy but HPV‐infected group, 33.33% in the CIN1 group, 42.55% in the CIN2 group, 57.58% in the CIN3 group, and 92.31% among cervical cancer patients (Figure 3D). Further evaluation of the correlation with vaginal microbiota revealed a significant decrease in alpha diversity with lower pH values, particularly when comparing vaginal microbiota from women with a pH greater than 5 to those below 4.5 (*p* = 0.000079, Figure 3E and Figure S2C). Low pH values strongly correlated with *Lactobacillus* species in the vaginal microbiota, as women in the pH < 4.5 and pH 4.5–5 groups were clustered in the same *Lactobacillus*‐dominated area (*p* = 0.001) (Figures 1B and 4F, Figure S2D, and Table 2). This finding is further supported by the identification of four genera that were differentially enriched among the three pH level groups, with *Lactobacillus* significantly enriched in the pH < 4.5 women's group and *Dialister* and *Anaerococcus* more abundant in the group of women with a pH > 5 (Figure 3G).

### 3.5 H_2_O_2_ Showed an Association With Cervical Dysplasia Stages and Microbiota Diversity

The percentage of women with H_2_O_2_ levels lower than 2 μmol/L gradually increased from 33.33% in the healthy group to 49.02% in the healthy but HPV‐positive group, 52.27% in the CIN2 group, and 69.23% in cervical cancer patients (Figure 4A). There was no significant difference in microbiota alpha diversity among women with different levels of H_2_O_2_ (Figure 4B and Figure S3A). However, the microbial beta diversity analysis revealed that most samples in the H_2_O_2_ above 4 μmol/L group clustered together in the *Lactobacillus crispatus* ‐dominated area. In contrast, samples in the H_2_O_2_ below 2 μmol/L group were primarily located in the *L. iners* and non‐*Lactobacillus*‐dominated microbiota area (*p* = 0.001, Figure 4C, Figure S3B and Table 2). The LEfSe analysis revealed that *Lactobacillus* was more enriched in the H_2_O_2_ above 4 μmol/L group, while *Actinobacteria* and *Aerococcus* were more abundant in the H_2_O_2_ below 2 μmol/L group (Figure S3C).

### 3.6 Women in the Elder Age Group Had Higher Microbiota Diversity and More Cervical Cancer Cases

All the cervical cases examined in our study were found in women aged above 30 years old. The mean age of the healthy HPV‐negative, healthy HPV‐positive, CIN1, CIN2, CIN3, and cervical cancer groups were 34.06, 38.13, 39.80, 38.30, 38.88, and 50.29, respectively (Figure 4D). Vaginal microbiota diversity increased with age, and women in the 45–75 years old age group exhibited significantly higher microbiota alpha diversity than those in the 18–30 years old age group (*p* = 0.036, Figure 4E). Additionally, the majority of women in the 45–75 years old age group clustered in the non‐*Lactobacillus*‐dominated area, while women below 30 years old tended to have vaginal microbiota that was *Lactobacillus*‐dominated (*p* = 0.001, Figures 1B and 4F and Table 2). The LEfSe analysis of bacterial abundance among different age groups revealed that *Lactobacillus* and *Moraxellaceae bacterium* genera were enriched in the 18–30 years old age group, while *Actinomyces* and *Burkholderiaceae* were significantly increased in the 45–75 age group (Figure 4G).

### 3.7 Vaginal Microbiota Diversity Is Influenced by Partner Number and Contraceptive Methods

In addition to the observed trends of higher microbiota diversity among women with higher dysplasia stages and HPV infection, we also found that partner number (1 vs. 2 or more) and contraceptive methods (contraception vs. condom vs. intrauterine device, IUD) influenced the beta diversity of the vaginal microbiota (Figures S4 and S5). Although the vaginal microbiota alpha diversity was comparable between women with different partner numbers and contraceptive methods (Figures S4A and S5A), the beta diversity analysis revealed that women with multiple partners and those using contraception methods such as condoms and IUDs clustered on the non‐*Lactobacillus*‐dominated side (Figure 1A, Figures S4B and S5B). Various bacteria were found to be differentially enriched in women with one partner versus multiple partners, with *Aquincola* and *Ureaplasma* being more abundant in women with IUDs than those without contraception (Figures S4D and S5D).

To explore the clinical significance of our data, we analyzed the diagnostic ability of clinical indices and vaginal microbiota species (*Lactobacillus* and the top 10 genera). The prediction values for HPV16/18 infection, pH > 5, H_2_O_2_ < 2 μmol/L, and age over 45 were scored at 69.70%, 75.20%, 59.50%, and 60.10%, respectively. Combining all these factors resulted in a prediction score of 81.90% (Figure S5E). Among the top three bacteria with the highest scores for predicting cervical dysplasia and cervical cancer were *Burkholderiaceae bacterium*, *Acinetobacter*, and *Prevotella*, with scores of 72.90%, 71.90%, and 70.00%, respectively (Figure S5F). When combining all the factors with *Burkholderiaceae bacterium* or an *Acinetobacter* proportion above 1.00%, the prediction values further increased to 82.90% and 84.30%, respectively (Figure S5G).

## 4 Discussion

This study covers distinct stages of cervical dysplasia and analyzes the association of multiple factors in the vaginal tract, including vaginal microbiota, HPV infection, pH value, H_2_O_2_ level, age, partner numbers, and contraceptive method. We demonstrated a significant increase in HPV 16 infection, a higher prevalence of pH > 5, a lower H_2_O_2_ level, and older ages among patients with cervical dysplasia and cancer compared to healthy individuals. Additionally, these factors influence the beta diversity of the vaginal microbiota, with fewer *Lactobacillus* species, especially *L. crispatus* , and more non‐*Lactobacillus* species, such as *Actinomyces* and *Burkholderiaceae*.

Many studies have reported a positive correlation between cervical cancer progression and high vaginal microbiota diversity [25, 26, 27]. Among all the clinical samples, we observed main clusters of *L. crispatus* ‐dominated, *L. iners* ‐dominated, and non‐*Lactobacillus*‐dominated vaginal microbiomes, which are similar to what has been observed among Swedish women and in other studies around the world [8, 28]. We also observed a decrease in *L. crispatus* ‐dominated vaginal microbiota as cervical dysplasia progresses, which is supported by other studies [2, 3, 17]. However, we demonstrated a higher prevalence of *Lactobacillus*‐dominated vaginal microbiota among women with different lesion grades compared to other reports, possibly due to geographic and ethnic variations [25, 29, 30]. This leads to non‐significant results in microbiota comparison among HPV‐infected and uninfected groups, as well as in different cervical dysplasia groups. Consistent with earlier studies, our data reported a high ratio of women infected with HPV16/18, which increased with the development of dysplasia stages. We identified a significant association between HPV infection, cervical dysplasia, and vaginal microbiota beta diversity.

Notably, we found that *Burkholderiaceae*, *Acinetobacter*, and *Streptococcus*, previously associated with preterm birth and sexually transmitted diseases, are increased in dysplasia and cancer patients compared to healthy, HPV‐negative individuals [31, 32]. Consistent with our findings, other groups have also observed higher levels of *Pelomonas* and *Ochrobactrum* among healthy individuals, which decreased among patients with dysplasia and cancer [33, 34]. Furthermore, *L. iners* is shown to co‐exist with other potentially harmful bacteria and be less effective in protecting against pathogen colonization, which might contribute to the onset of vaginal dysbiosis [35, 36, 37]. A study conducted in a Korean cohort showed the enrichment of *L. iners* , *A. vaginae* , and *G. vaginalis* with a concomitant paucity of *L. crispatus* in CIN2 women compared to CIN1 women, and a similar trend can be observed from our data [38].

We found a strong association between increased pH and cervical dysplasia. Lactic acid‐producing microbes, primarily *Lactobacillus* species in the women's vaginal tract, create an acidic environment with a pH of ≤ 4.5 [39]. Lactic acid at pH 4.5 has been shown to protect against various bacterial vaginosis‐associated species, including *Gardnerella vaginalis* and *Atopobium vaginae* , without affecting the viability of *Lactobacillus* [40]. Our data is consistent with these findings, as women with a pH > 5 exhibited higher microbiota diversity and lower abundance of *Lactobacillus* than women with lower pH values. Furthermore, our data supported the enrichment of anaerobic bacteria, such as *Dialister* and *Anaerococcus*, in women with a pH > 5. *Dialister* has been reported as a biomarker for high‐risk HPV infection [41]. *Anaerococcus* has been implicated in a higher frequency and severity of HPV infection, potentially promoting the progression of pre‐cancerous and cancerous cervical lesions [42].

Bacteria, such as *Burkholderiaceae*, *Acinetobacter*, and *Streptococcus*, which are related to cancer cases, as well as *Ochrobactrum* and *Anaerococcus*, found to be significantly altered in certain groups, have also been identified in the gut microbiota [43, 44]. Moreover, crosstalk between gut and vaginal microbiota can occur via short chain fatty acid or immunological effects [45]. Other factors may influence vaginal microbiota both directly and indirectly through gut microbiota.

H_2_O_2_ has been suggested as an antimicrobial factor, and the prevalence of H_2_O_2_‐generating *Lactobacillus* species is found to be lower among women with bacterial vaginosis [46, 47, 48]. Our study demonstrated lower microbiota beta diversity with higher enrichment of *L. crispatus* , but not *L. iners* , in the group with high levels of H_2_O_2_. This finding is reasonable since *L. iners* has been reported to lack the molecular and cellular machinery to generate H_2_O_2_ [49]. Furthermore, our data is supported by the observation that the group with H_2_O_2_ levels > 4 μmol/L is distributed around the *L. crispatus* ‐dominated area and is distinct from the group of women with lower H_2_O_2_ levels.

A healthy vaginal microbiota is generally stable during the reproductive age, with fluctuations synchronizing with the menstrual cycle [50, 51]. The abundance of *Lactobacillus* species significantly decreases as estrogen declines in menopausal women [20, 50, 52]. In our study, which included participants ranging from 18 to 75 years old, we demonstrated that microbiota diversity increases in women above 45 years old. Additionally, age itself is a risk factor for cervical cancer, as we observed a positive correlation between age and cervical cancer in our data. When considering the microbiota, women aged above 45 with a low abundance of *Lactobacillus* have the highest risk for cervical dysplasia.

Sexual behavior has been shown to influence the composition of the vaginal microbiota [53]. A cross‐sectional study reported that women who engaged in unprotected sexual activity and had more sexual partners were more likely to be dominated by *L. iners* [54]. In a longitudinal cohort study, users of combined oral contraceptive pills were more likely to have a stable microbiota over time with *Lactobacillus* dominance compared to non‐users, specifically among White women but not African American women [55]. Hormonal contraceptive use increased the abundance of total vaginosis‐associated bacteria in the vaginal microbiota of Black women but not White women [56]. Additionally, in Kenyan women, postpartum vaginal bacterial diversity was significantly higher in hormonal contraceptive users compared to non‐users [57]. However, studies investigating sexual behavior and contraceptive methods in the Chinese population, particularly in relation to vaginal microbiota, are scarce. In our study, we demonstrated that using intrauterine devices and condoms significantly influenced the beta diversity of the vaginal microbiota among Chinese women. Furthermore, we found that combining vaginal microbiota analysis with current diagnostic testing, including HPV16/18 infection, pH > 5, H_2_O_2_ < 2 μmol/L, and age over 45, could enhance the potential for diagnosing cervical dysplasia and cervical cancer. Further evaluation and validation of incorporating these bacteria into cancer diagnosis are very important. More research is needed to elucidate the underlying mechanisms of these different effects observed in different racial groups.

Our study is limited because the samples were obtained from a single location. However, this limitation also eliminates potential influences caused by other confounding factors that may alter the microbiota. Additionally, the cervical cancer group in our cohort is relatively small, which may affect the statistical analysis when comparing microbiota data. Lastly, we obtained metagenomic information through 16S rRNA gene sequencing, which is known to have lower resolution at the species level. We performed manual BLAST searches to identify major bacterial species to address this limitation. Although these limitations exist, our data provides a foundation for future studies that involve a larger number of cervical cancer patients or utilize longitudinal cohorts to validate the correlation between cervical cancer development, vaginal microbiota, and other clinical factors.

## 5 Conclusion

In conclusion, we discovered a close association between vaginal microbiota and cervical dysplasia stages, which is influenced by factors such as vaginal HPV infection, pH, H_2_O_2_ level, partner number, and contraceptive methods. These findings underscore the significance of considering the vaginal microbiota within the cancer microenvironment.

## Author Contributions

**Liqin Cheng:** conceptualization (equal), data curation (equal), formal analysis (equal), methodology (equal), resources (equal), software (equal), validation (equal), visualization (equal), writing – original draft (equal), writing – review and editing (equal). **Chunmei Yan:** conceptualization (equal), data curation (equal), methodology (equal), project administration (equal), resources (equal), writing – original draft (equal), writing – review and editing (equal). **Yongxia Yang:** methodology (equal), project administration (equal), resources (equal), writing – original draft (equal), writing – review and editing (equal). **Fanzhen Hong:** project administration (equal), resources (equal), writing – review and editing (equal). **Juan Du:** conceptualization (equal), funding acquisition (equal), investigation (equal), project administration (equal), resources (equal), supervision (equal), visualization (equal), writing – original draft (equal), writing – review and editing (equal).

## Ethics Statement

All participation was voluntary and anonymous, and written informed consent was obtained. The study was approved by the Regional Ethical Board at the Second Hospital of Shandong University, China.

## Conflicts of Interest

The authors declare no conflicts of interest.

## Supporting information


**Table S1.** Metadata with the clinical test results for all the sequenced samples.

**Figure S1.** Vaginal microbial alpha diversity of women with different microbiota composition and cancer progression. (A) Vaginal microbial alpha diversity based on Shannon, Chao1, and Faith’s PD index of women with different microbiota composition. (B) Vaginal microbial alpha diversity based on Shannon, Chao1, and Faith’s PD index among healthy individuals with and without HPV infection and dysplasia and cancer patients. The data were presented with median values and 1.5 times the interquartile range (IQR). Statistical significance between the groups was tested by Kruskal–Wallis with Dunn’s test as a post hoc test. (C) Principal coordinates analysis (PCoA) of vaginal microbiota among healthy individuals with and without HPV infection and dysplasia and cancer patients based on Bray–Curtis distance.

**Figure S2.** Vaginal microbiota diversity among women with different HPV infection status and pH values. (A) Vaginal microbial alpha diversity based on Shannon, Chao1, and Faith’s PD index among women with different HPV infection status. Data were presented with median values and 1.5 times the IQR. Statistical significance between the groups was tested by the Kruskal–Wallis test with Dunn’s test as a post hoc test. (B) PCoA of vaginal microbiota among women with different HPV infection statuses based on Bray–Curtis distance. (C) Vaginal microbial alpha diversity based on Shannon, Chao1, and Faith’s PD index of women with different pH values. The data were presented with median values and 1.5 times the IQR. Statistical significance between the groups was tested by the Kruskal–Wallis test with Dunn’s test as a post hoc test. ***p < 0.001, **p < 0.01. (D) PCoA of vaginal microbiota among women with different pH values based on Jaccard distance.

**Figure S3.** Vaginal microbiota diversity of women with different H_2_O_2_ levels and age groups. (A) Vaginal microbial alpha diversity based on Shannon, Chao1, and Faith’s PD index of women with different H_2_O_2_ levels. The data were presented with median values and 1.5 times the IQR. Statistical significance between the groups was tested by the Kruskal–Wallis test with Dunn’s test as a post hoc test. (B) PCoA of vaginal microbiota among women with different H_2_O_2_ levels based on Jaccard distance. (C) LEfSe analysis showed significantly different expressed microbes among women with different H_2_O_2_ levels. Only taxa with linear discriminant analysis (LDA) scores over 2 were presented. (D) Vaginal microbial alpha diversity based on Shannon, Chao1, and Faith’s PD index of women with different age groups. The data were presented with median values and 1.5 times the IQR. Statistical significance between the groups was tested by the Kruskal–Wallis test with Dunn’s test as a post hoc test. (E) PCoA of vaginal microbiota among women with different age groups based on Jaccard distance.

**Figure S4.** The association of vaginal microbiota with the number of sexual partners. (A) Vaginal microbial alpha diversity analysis based on Simpson, Shannon, Chao1, and Faith’s PD index of women with one or more partners. The data were presented with median values and 1.5 times the IQR. Statistical significance between the groups was tested by the Kruskal–Wallis test with Dunn’s test as a post hoc test. (B) PCoA of vaginal microbiota presented by one or more partner numbers based on Jaccard distance. (C) PCoA of vaginal microbiota among women with one or more partners based on Bray–Curtis distance. (D) LEfSe analysis showed significantly different expressed microbes among women with one or more partner numbers. Only taxa with linear discriminant analysis (LDA) scores over 2 were presented.

**Figure S5.** The association of vaginal microbiota with contraceptive methods. (A) Vaginal microbial alpha diversity based on Simpson, Shannon, Chao1, and Faith’s PD index of women with different contraceptive methods. The data were presented with median values and 1.5 times the IQR. Statistical significance between the groups was tested by the Kruskal–Wallis test with Dunn’s test as a post hoc test. (B) PCoA of vaginal microbiota presented by different contraception methods based on Jaccard distance. (C) PCoA of vaginal microbiota among women with different contraceptive methods based on Bray–Curtis distance. (D) LEfSe analysis showed significantly different expressed microbes among women with different contraception methods. Only taxa with linear discriminant analysis (LDA) scores over 2 were presented. (E) Receiver operating characteristics (ROC) curves of HPV16/18 infection, pH > 5, H_2_O_2_ < 2 μmol/L, and age > 45 for predicting cervical cancer progression. (F) ROC curves of *Burkholderiaceae bacterium*, *Acinetobacter*, and *Prevotella* for predicting cervical cancer progression. (G) ROC curves of *Burkholderiaceae bacterium* > 1%, *Acinetobacter* > 1%, and *Prevotella* > 1% with all indexes in (E) to predict cervical cancer progression.

## Data Availability

All data generated or analyzed in this study are included in this article. The sequencing reads have been submitted to the NCBI Sequence Read Archive (SRA) under the accession number PRJNA993994.
