# Supplementary figures and images for: Exploring the Clinical Signatures of Cervical Dysplasia Patients and Their Association With Vaginal Microbiota
Source: Cancer Med. 2024 Dec 6;13(23):e70440. doi: 10.1002/cam4.70440 (PMC11622153; doi:10.1002/cam4.70440)

Figure S1

A

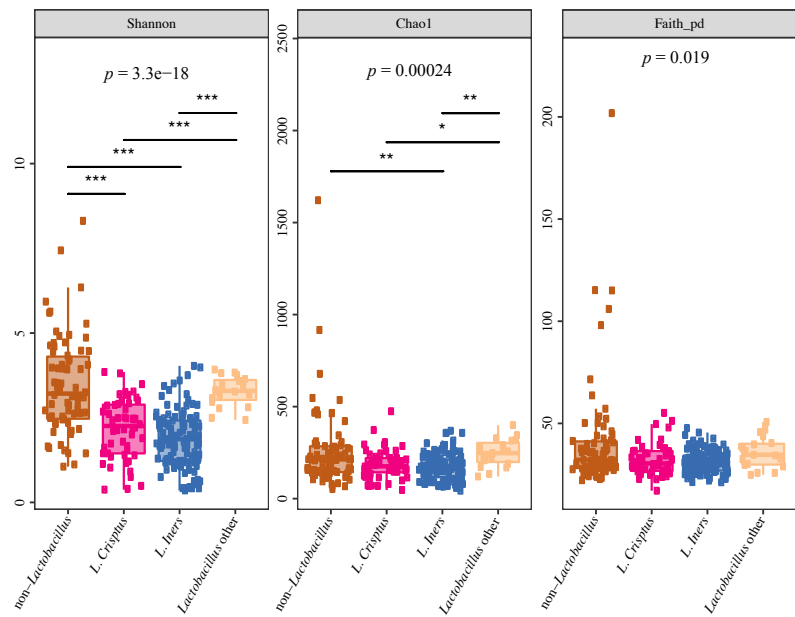

B

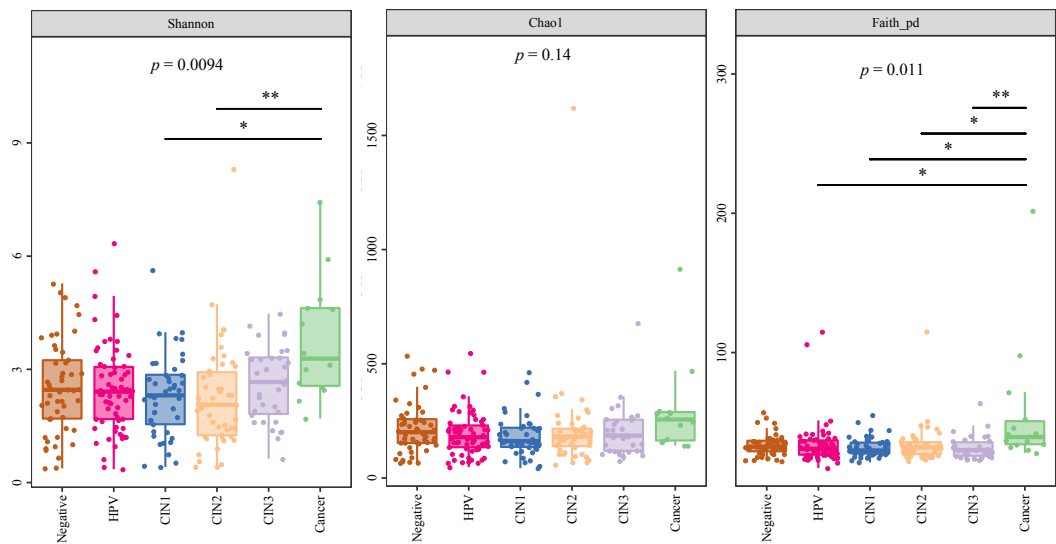

C

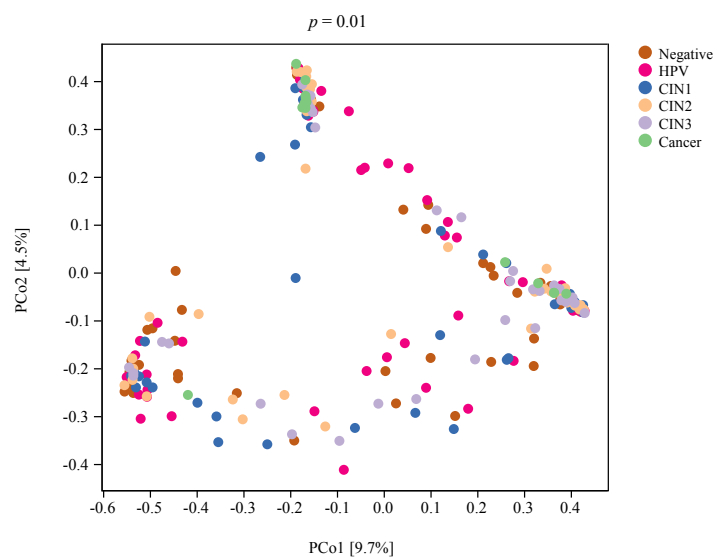

Supplement: Supplementary file 2 — Figure S1. Vaginal microbial alpha diversity of women with different microbiota composition and cancer progression. (A) Vaginal microbial alpha diversity based on Shannon, Chao1, and Faith’s PD index of women with different microbiota composition. (B) Vaginal microbial alpha diversity based on Shannon, Chao1, and Faith’s PD index among healthy individuals with and without HPV infection and dysplasia and cancer patients. The data were presented with median values and 1.5 times the interquartile range (IQR). Statistical significance between the groups was tested by Kruskal–Wallis with Dunn’s test as a post hoc test. (C) Principal coordinates analysis (PCoA) of vaginal microbiota among healthy individuals with and without HPV infection and dysplasia and cancer patients based on Bray–Curtis distance. [file CAM4-13-e70440-s004.pdf]

Figure S2

A

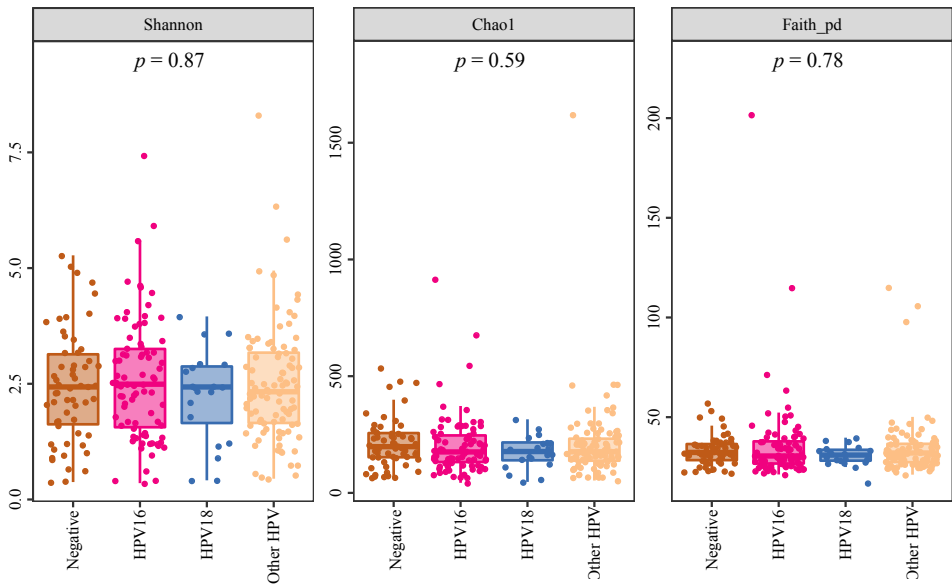

B

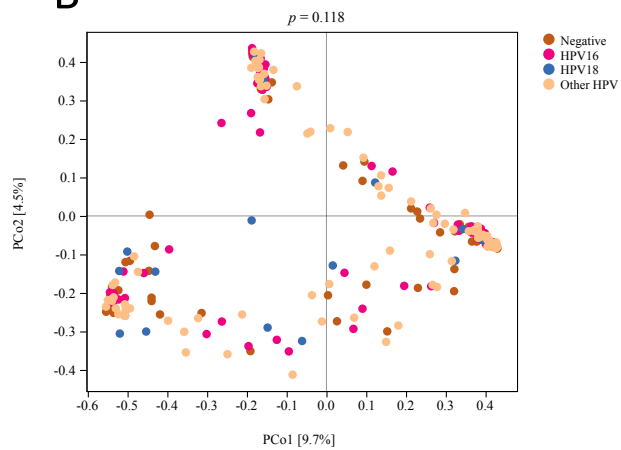

C

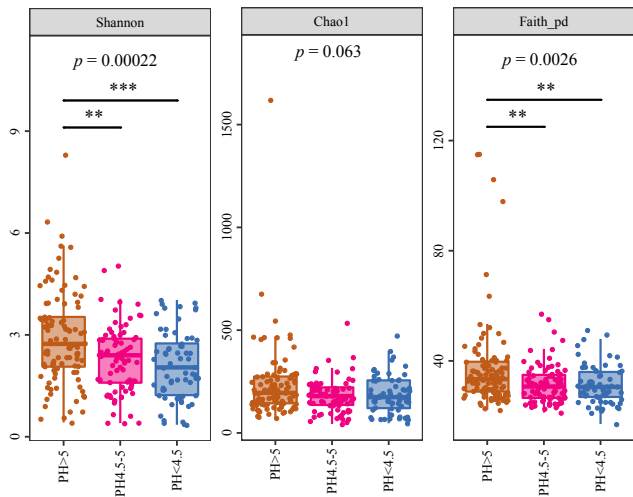

D

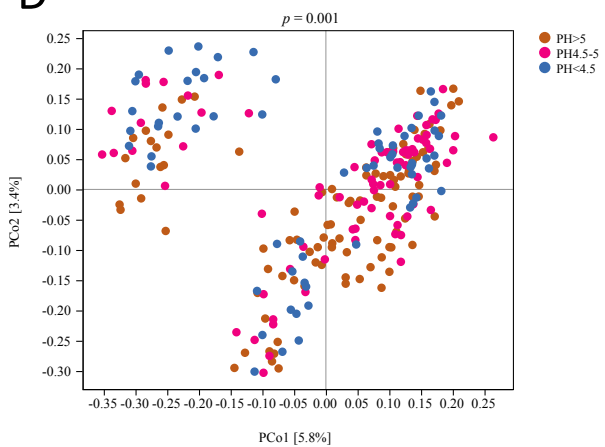

Supplement: Supplementary file 3 — Figure S2. Vaginal microbiota diversity among women with different HPV infection status and pH values. (A) Vaginal microbial alpha diversity based on Shannon, Chao1, and Faith’s PD index among women with different HPV infection status. Data were presented with median values and 1.5 times the IQR. Statistical significance between the groups was tested by the Kruskal–Wallis test with Dunn’s test as a post hoc test. (B) PCoA of vaginal microbiota among women with different HPV infection statuses based on Bray–Curtis distance. (C) Vaginal microbial alpha diversity based on Shannon, Chao1, and Faith’s PD index of women with different pH values. The data were presented with median values and 1.5 times the IQR. Statistical significance between the groups was tested by the Kruskal–Wallis test with Dunn’s test as a post hoc test. ***p < 0.001, **p < 0.01. (D) PCoA of vaginal microbiota among women with different pH values based on Jaccard distance. [file CAM4-13-e70440-s003.pdf]

Figure S3

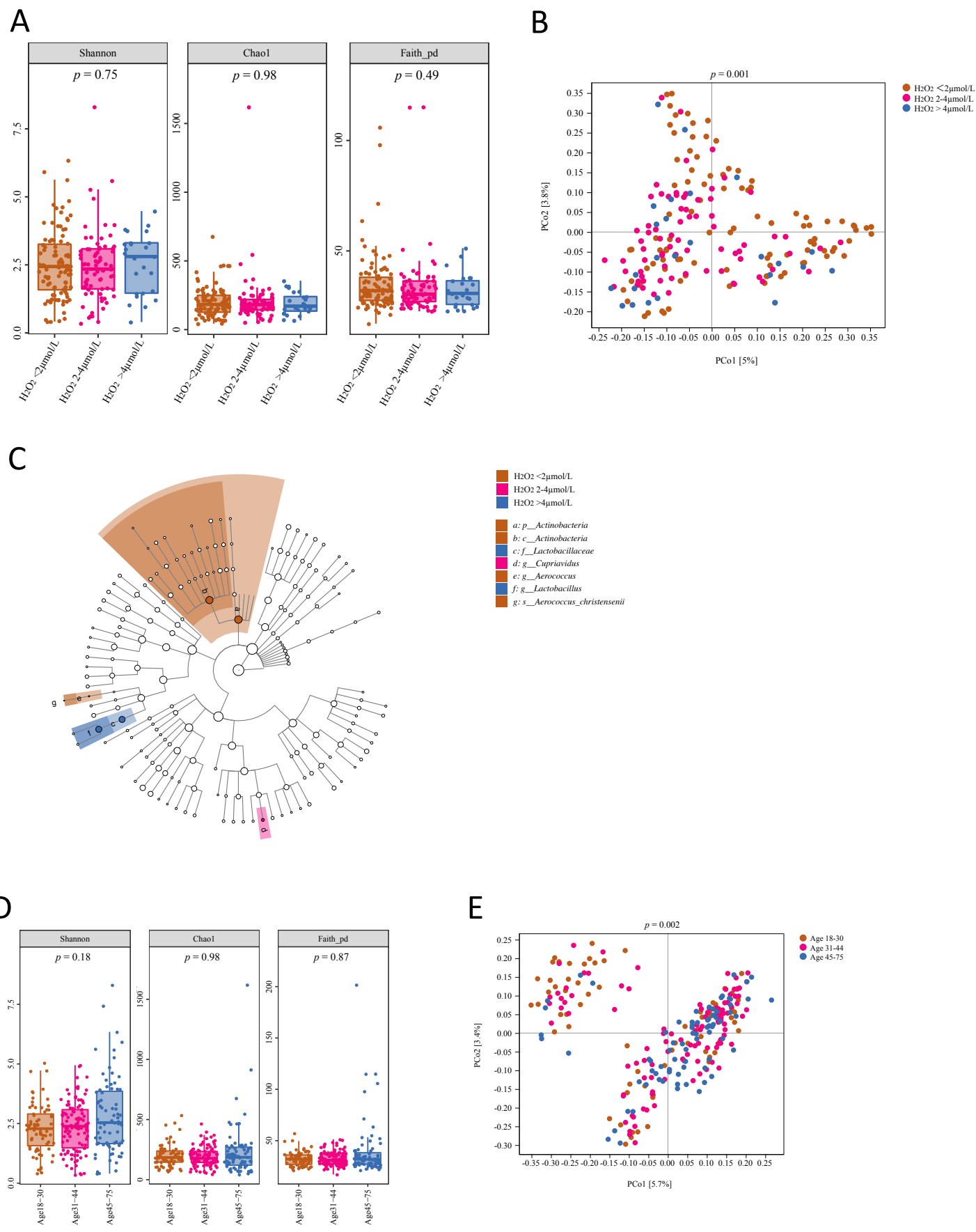

Supplement: Supplementary file 4 — Figure S3. Vaginal microbiota diversity of women with different H2O2 levels and age groups. (A) Vaginal microbial alpha diversity based on Shannon, Chao1, and Faith’s PD index of women with different H2O2 levels. The data were presented with median values and 1.5 times the IQR. Statistical significance between the groups was tested by the Kruskal–Wallis test with Dunn’s test as a post hoc test. (B) PCoA of vaginal microbiota among women with different H2O2 levels based on Jaccard distance. (C) LEfSe analysis showed significantly different expressed microbes among women with different H2O2 levels. Only taxa with linear discriminant analysis (LDA) scores over 2 were presented. (D) Vaginal microbial alpha diversity based on Shannon, Chao1, and Faith’s PD index of women with different age groups. The data were presented with median values and 1.5 times the IQR. Statistical significance between the groups was tested by the Kruskal–Wallis test with Dunn’s test as a post hoc test. (E) PCoA of vaginal microbiota among women with different age groups based on Jaccard distance. [file CAM4-13-e70440-s005.pdf]

Figure S4

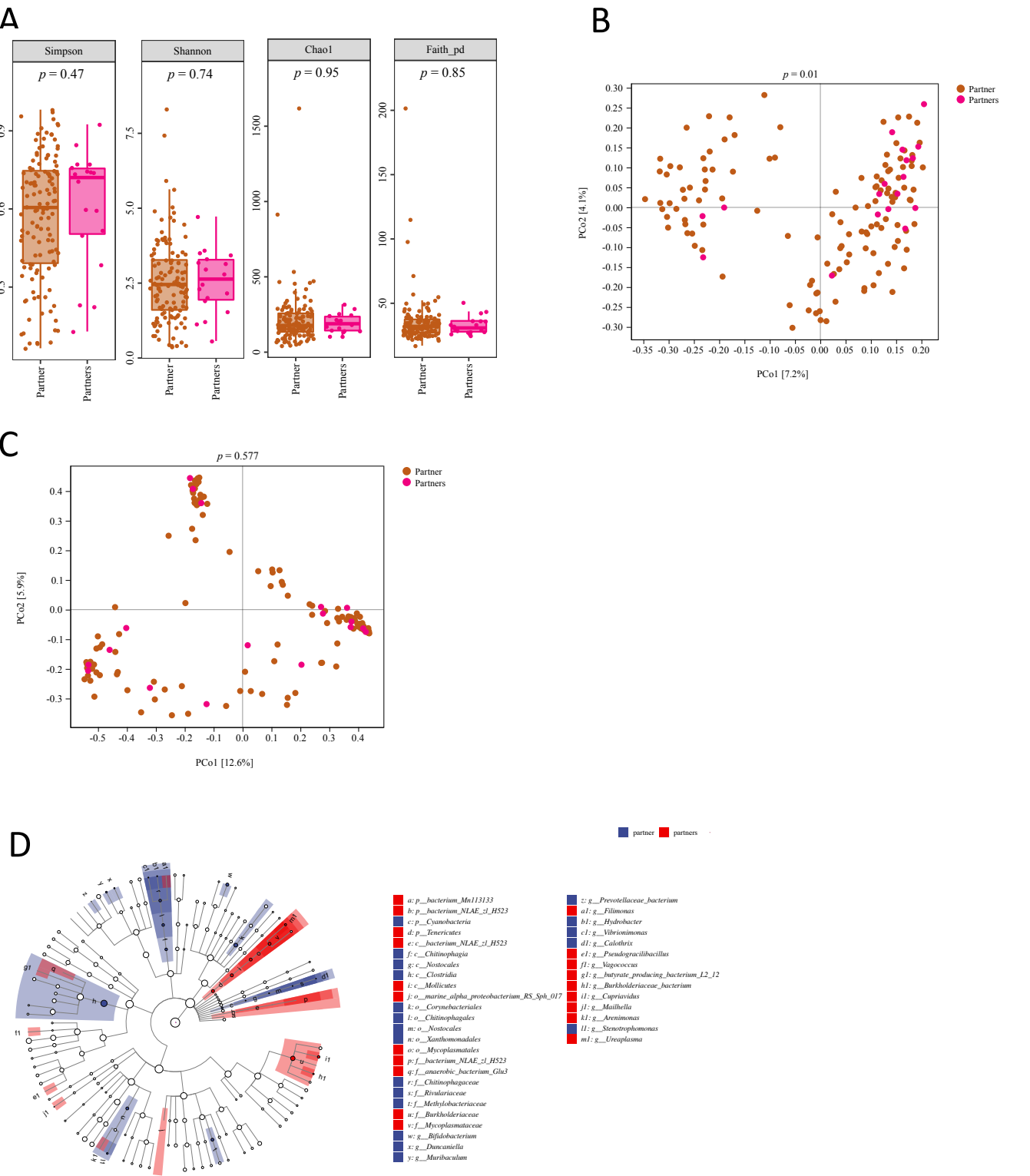

Supplement: Supplementary file 5 — Figure S4. The association of vaginal microbiota with the number of sexual partners. (A) Vaginal microbial alpha diversity analysis based on Simpson, Shannon, Chao1, and Faith’s PD index of women with one or more partners. The data were presented with median values and 1.5 times the IQR. Statistical significance between the groups was tested by the Kruskal–Wallis test with Dunn’s test as a post hoc test. (B) PCoA of vaginal microbiota presented by one or more partner numbers based on Jaccard distance. (C) PCoA of vaginal microbiota among women with one or more partners based on Bray–Curtis distance. (D) LEfSe analysis showed significantly different expressed microbes among women with one or more partner numbers. Only taxa with linear discriminant analysis (LDA) scores over 2 were presented. [file CAM4-13-e70440-s001.pdf]

Figure S5

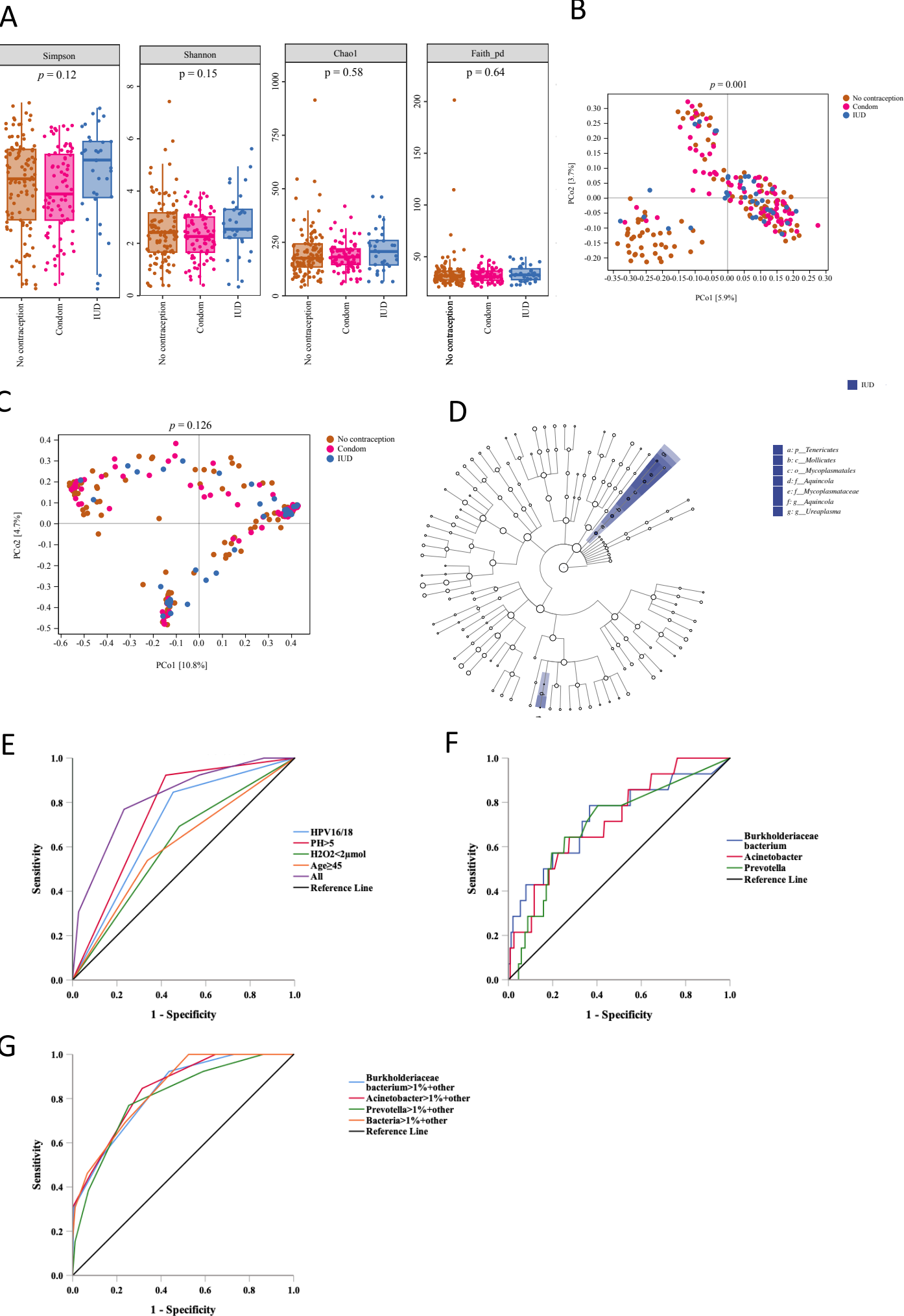

Supplement: Supplementary file 6 — Figure S5. The association of vaginal microbiota with contraceptive methods. (A) Vaginal microbial alpha diversity based on Simpson, Shannon, Chao1, and Faith’s PD index of women with different contraceptive methods. The data were presented with median values and 1.5 times the IQR. Statistical significance between the groups was tested by the Kruskal–Wallis test with Dunn’s test as a post hoc test. (B) PCoA of vaginal microbiota presented by different contraception methods based on Jaccard distance. (C) PCoA of vaginal microbiota among women with different contraceptive methods based on Bray–Curtis distance. (D) LEfSe analysis showed significantly different expressed microbes among women with different contraception methods. Only taxa with linear discriminant analysis (LDA) scores over 2 were presented. (E) Receiver operating characteristics (ROC) curves of HPV16/18 infection, pH > 5, H2O2 < 2 μmol/L, and age > 45 for predicting cervical cancer progression. (F) ROC curves of Burkholderiaceae bacterium, Acinetobacter, and Prevotella for predicting cervical cancer progression. (G) ROC curves of Burkholderiaceae bacterium > 1%, Acinetobacter > 1%, and Prevotella > 1% with all indexes in (E) to predict cervical cancer progression. [file CAM4-13-e70440-s006.pdf]
